# VCP Associated Inclusion Body Myopathy and Paget Disease of Bone Knock-In Mouse Model Exhibits Tissue Pathology Typical of Human Disease

**DOI:** 10.1371/journal.pone.0013183

**Published:** 2010-10-05

**Authors:** Mallikarjun Badadani, Angèle Nalbandian, Giles D. Watts, Jouni Vesa, Masashi Kitazawa, Hailing Su, Jasmin Tanaja, Eric Dec, Douglas C. Wallace, Jogeshwar Mukherjee, Vincent Caiozzo, Matthew Warman, Virginia E. Kimonis

**Affiliations:** 1 Department of Pediatrics, University of California Irvine, Irvine, California, United States of America; 2 Department of Orthopedic Surgery, Children's Hospital Boston, Harvard Medical School, Boston, Massachusetts, United States of America; 3 Department of Neurobiology and Behavior, University of California Irvine, Irvine, California, United States of America; 4 Center for Molecular and Mitochondrial Medicine and Genetics, University of California Irvine, Irvine, California, United States of America; 5 Departments of Ecology and Evolutionary Biology, University of California Irvine, Irvine, California, United States of America; 6 Department of Biological Chemistry, University of California Irvine, Irvine, California, United States of America; 7 Department of Psychiatry & Human Behavior, University of California Irvine, Irvine, California, United States of America; 8 Departments of Physiology and Biophysics, and Orthopedics, University of California Irvine, Irvine, California, United States of America; 9 Department of Genetics, Children's Hospital Boston, Harvard Medical School, Boston, Massachusetts, United States of America; 10 Department of Cell Biology and Biochemistry, School of Medicine, Health Policy and Practice, University of East Anglia, Norwich, Norfolk, United Kingdom; Johns Hopkins School of Medicine, United States of America

## Abstract

Dominant mutations in the valosin containing protein (VCP) gene cause inclusion body myopathy associated with Paget's disease of bone and frontotemporal dementia (IBMPFD). We have generated a knock-in mouse model with the common R155H mutation. Mice demonstrate progressive muscle weakness starting approximately at the age of 6 months. Histology of mutant muscle showed progressive vacuolization of myofibrils and centrally located nuclei, and immunostaining shows progressive cytoplasmic accumulation of TDP-43 and ubiquitin-positive inclusion bodies in quadriceps myofibrils and brain. Increased LC3-II staining of muscle sections representing increased number of autophagosomes suggested impaired autophagy. Increased apoptosis was demonstrated by elevated caspase-3 activity and increased TUNEL-positive nuclei. X-ray microtomography (uCT) images show radiolucency of distal femurs and proximal tibiae in knock-in mice and uCT morphometrics shows decreased trabecular pattern and increased cortical wall thickness. Bone histology and bone marrow derived macrophage cultures in these mice revealed increased osteoclastogenesis observed by TRAP staining suggestive of Paget bone disease. The VCP^R155H/+^ knock-in mice replicate the muscle, bone and brain pathology of inclusion body myopathy, thus representing a useful model for preclinical studies.

## Introduction

Inclusion body myopathy associated with Paget's disease of bone and frontotemporal dementia (IBMPFD, OMIM 167320) is characterized by progressive muscle weakness, bone deformities and extensive neuro-degeneration [1]. Muscle weakness and atrophy of the pelvic and shoulder girdle muscles is seen in 82% of patients at the mean age of 42 years [1]–[4]. Disease progression is characterized by generalized muscle weakness resulting in respiratory and cardiac failure in the later stages [5]. The diagnosis of IBMPFD muscle disease is based on skeletal muscle weakness, and the presence of rimmed vacuoles and inclusion bodies in the muscle [1], [6], [7]. These inclusions have been shown to be positive for the TAR DNA binding protein-43 (TDP-43) and ubiquitin antibodies [8]. Although the specific function of TDP-43 is not clear, its cytoplasmic accumulation in skeletal muscle tissue has been used as a disease marker in human myopathies [8]. Studies in patient myoblasts have revealed vacuoles and increased LC3 and autophagosome formation suggestive of impaired autophagy [9].

Typically Paget's disease of bone (PDB) seldom affects individuals under the age of 50 years. However, PDB is observed in 49% of IBMPFD patients [1], [4] and it typically begins in the 30 s to 40 s, the mean age of onset being 42 years [10]. PDB is caused by excessive osteoclastic activity and susceptibility to deformities like bowing and fractures. It involves focal areas of increased bone turnover that typically leads to spine and/or hip pain and deformity of the long bones causing pathological fractures on occasion. The most commonly involved bones are the skull, vertebrae and pelvis. The diagnosis of PDB is based on elevated concentrations of serum alkaline phosphatase (ALP), urine pyridinoline (PYD) and deoxypyridinoline (DPD), as well as on skeletal radiographs or radionuclide scans.

Frontotemporal dementia (FTD) comprises about 3% of dementias among the elderly [11]–[13]. In IBMPFD, premature FTD is observed in 27% of patients with an average age of onset in the mid 50 s [2]. It is characterized by dysnomia, dyscalculia, comprehension deficit, paraphasic errors, and relative preservation of memory. In later stages, patients have auditory comprehension deficits for even one-step commands, and exhibit alexia, and agraphia [1], [4]. Affected individuals die from progressive muscle weakness, as well as from cardiac and respiratory failure typically in their 40 s to 60 s [1]–[3].

Molecular genetic studies revealed that IBMPFD is caused by mutations in the Valosin Containing Protein (VCP) gene [7], and thus far 19 disease mutations have been identified (for review see [6], [14]). VCP belongs to the family of type II ATPase associated with variety of cellular activities (AAA) having two ATPase domains [15]–[19]. It has been suggested to be involved in a number of cellular activities, including homotypic membrane fusion, transcription activation, nuclear envelope reconstruction, post-mitotic organelle reassembly, cell cycle control, apoptosis and endoplasmic reticulum associated degradation of proteins (ERAD) [20]–[23]. Disease mutations cluster in the N-terminal domain, which is involved in ubiquitin-binding and protein-protein interactions [24], [25]. Most of these mutated residues causing IBMPFD are three-dimensionally located in close proximity and potentially interact with each other [26]. These findings suggest that these residues bind to several different adapter proteins enabling VCP to target specific substrates for proteasomal degradation [27], [28].

To be able to study in vivo effects of identified VCP mutations, as well as to understand the pathogenesis of IBMPFD, mouse models have been previously generated. Both human and mouse VCP proteins consist of 806 amino acids, and the mouse protein differs only by one amino acid residue at position 684 when compared to the corresponding human protein [29]. Targeted homozygous deletion of VCP by Cre-loxP technology was reported to result in early embryonic lethality [30] suggesting an important role for the intact VCP expression in the development of mouse embryos. In contrast, hemizygotes lacking one VCP allele were apparently indistinguishable from their wild-type littermates. On the other hand, transgenic mice over-expressing the most common human IBMPFD mutation, R155H, under the regulation of a muscle creatine kinase promoter demonstrated progressive muscle weakness in a dose-dependent manner starting at 6 months of age. These mutant mice demonstrated variation in muscle fiber size, increase in endomysial connective tissue, and ubiquitin positive sarcoplasmic and myonuclear vacuoles. They also showed inclusions and small linear basophilic rimmed cracks rather than the classic rimmed vacuoles [31]. Recently, Custer et al. (2010) [32] reported a transgenic mouse overexpressing mutant forms of VCP. These mice manifest muscle weakness, pathology characteristic of inclusion body myopathy including blue rimmed vacuoles and TDP-43 pathology. Radiological examination of the skeleton revealed focal lytic and sclerotic regions in the vertebrae and femur. Additionally, the brain revealed widespread TDP-43 lesions and the mice also exhibited abnormalities in behavioral testing.

Here, we report the generation and characterization of the VCP^R155H/+^ knock-in mouse models for IBMPFD. We decided to generate a knock-in model, since these mice express the mouse VCP gene at endogenous level and are therefore believed to represent a better model to study the pathology of the human disease and to develop targeted molecular treatment. Our preliminary results presented here are based on the analyses of heterozygous mice for R155H. Our analyses revealed that mutant mice show progressive muscle weakness starting at approximately 6 months of age. They also demonstrate progressive accumulation of ubiquitin and TDP-43 positive inclusion bodies in the muscle and brain. Muscle fibers also showed central nucleation and altered sarcomere ultrastructure, as well as increased autophagy and apoptosis. MicroCT analyses show increased bone activity, cortical thickness and bone histology showed osteoclastogenesis and increased TRAP (Tartrate Resistant Acid Phosphatase) staining seen in Paget-like bone lesions. These results suggest that the generated mice replicate the phenotype of human disease, and therefore can be used to study the pathological and molecular mechanisms underlying IBMPFD.

## Results

### Generation of VCP^R155H/+^ knock-in mice

**Figure 1:**
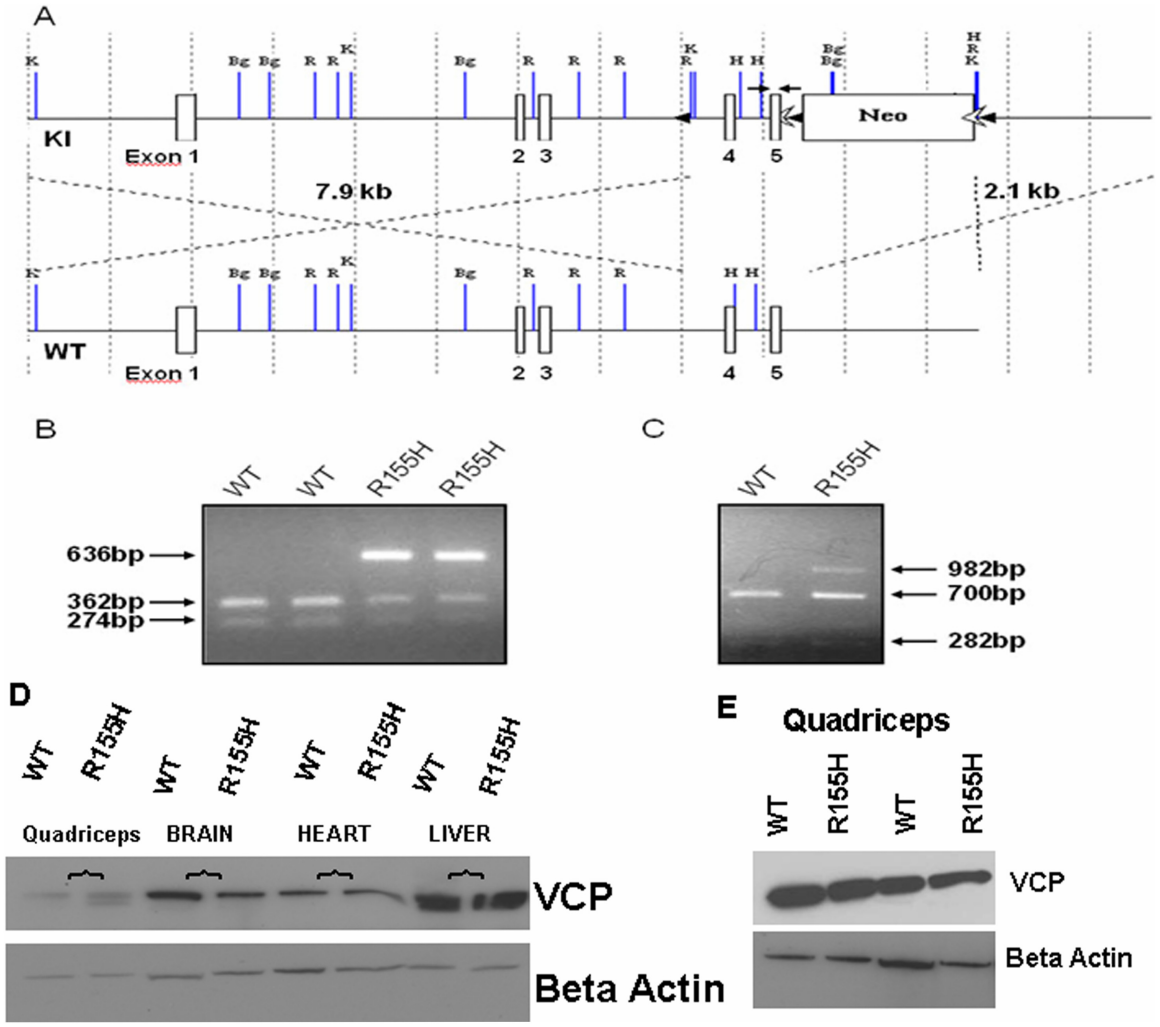
Generation of the VCP^R155H/+^ knock-in mice. (A) Schematic drawing of the R155H targeting strategy of the knock-in (KI) allele. The top line shows the knock-in (KI) allele and the below line depicts the wild-type (WT) allele. The localizations of exons 1 through 5 are numbered. Localizations of the 5′ (7.9 kb) and 3′ (2.1 kb) targeting sequences are indicated by dashed lines. Neomycin-cassette is marked by Neo, flanked by FRT sites and the restriction enzyme sites are indicated as follows: K  =  Kpn I, Bg  =  Bgl II, R  =  EcoR I, H  =  Hind III. Black arrow heads flanking Neo cassette show LoxP sites and white arrow heads show FRT sites. Black arrows flanking exon 5 indicate the locations of primers used in genotyping. (B) PCR genotyping of isolated from mouse tail DNA and digested by Msp I produces 274 bp and 362 bp in wild-type, while mutant allele gives three fragments of 636 bp, 274 bp and 362 bp. (C) RT-PCR-Msp I digestion analysis of mRNA isolated from mouse quadriceps shows similar expression levels. Wild-type allele produces 700 bp and 282 bp fragments, and mutant allele produces a 982 bp fragment. Fragment sizes are shown on the right and genotypes above. (D) VCP expression by western immunoblot in tissues: quadriceps, brain, heart and liver in wild type compared with knock-in mouse model shows similar expression levels. (E) VCP expression by Western immunoblot in quadriceps from wild type compared with knock-in mouse model. Beta actin was used as a loading control for these western blot analyses.

To generate a mouse model for human IBMPFD disease, we created knock-in mice that are heterozygous for the most common VCP disease mutation R155H through a Neo cassette insertion using 129/SvEv mice (**Figure 1A**). These mice were back-crossed more than six times with C57BL/6 strain before experiments were done to make sure that the majority (>98%) of the genetic background of generated mice was of C57BL/6 origin. C57BL/6 mice are also considered more suitable for behavioral testing due to their higher activity level. The heterozygous mutation did not affect the fertility or viability of mice since mutant and wild-type mice were born in Mendelian ratio. The genotypes of all mice were determined by PCR and RT-PCR encompassing the mutation site, and MspI digestion. PCR genotyping and digestion by MspI produced 274 bp and 362 bp fragments in wild-type while the mutant alleles produced 200 and 282 bp fragments while the mutant alleles gave 3 fragments of 636 bp, 274 bp, and 362 bp (**Figure 1B**). RT-PCR analysis of the mutant mouse tissue revealed that the mutant allele is expressed in the knock-in mice. The MspI digestion of the PCR amplified wild-type allele resulted in 700 bp and 282 bp fragments, whereas the mutant allele produced a fragment of 982 bp lacking the recognition site for MspI (**Figure 1C**). Western blot analysis of wild type and mutant quadriceps, brain, heart and liver tissues revealed similar VCP expression levels (**Figure 1D,E**). VCP expression by Q-RT-PCR analyses demonstrated no statistically significant differences between wild type (AVG ΔCt = 5.65) and R115H knock-in (AVG ΔCt = 5.46) mice with a 95% CI (p = 0.97).

The weight of wild-type and mutant mice were measured weekly from 3 weeks to 15 months of age. Measurements demonstrated that the weight of heterozygote mutant mice developed normally when compared to their wild-type littermates and other behavior including grooming, gait, and general activity was indistinguishable from their wild-type littermates; however, seizures were noted in up to 15% of the mice.

### Progressive muscle weakness in the VCP^R155H/+^ mouse model

Progressive muscle weakness is one of the primary symptoms in human IBMPFD patients [2]. Therefore, muscle strength as well as motor coordination and fatigue were measured between ages 3 and 15 months in 3-month intervals using grip strength test and a rotarod analysis, respectively.

**Figure 2:**
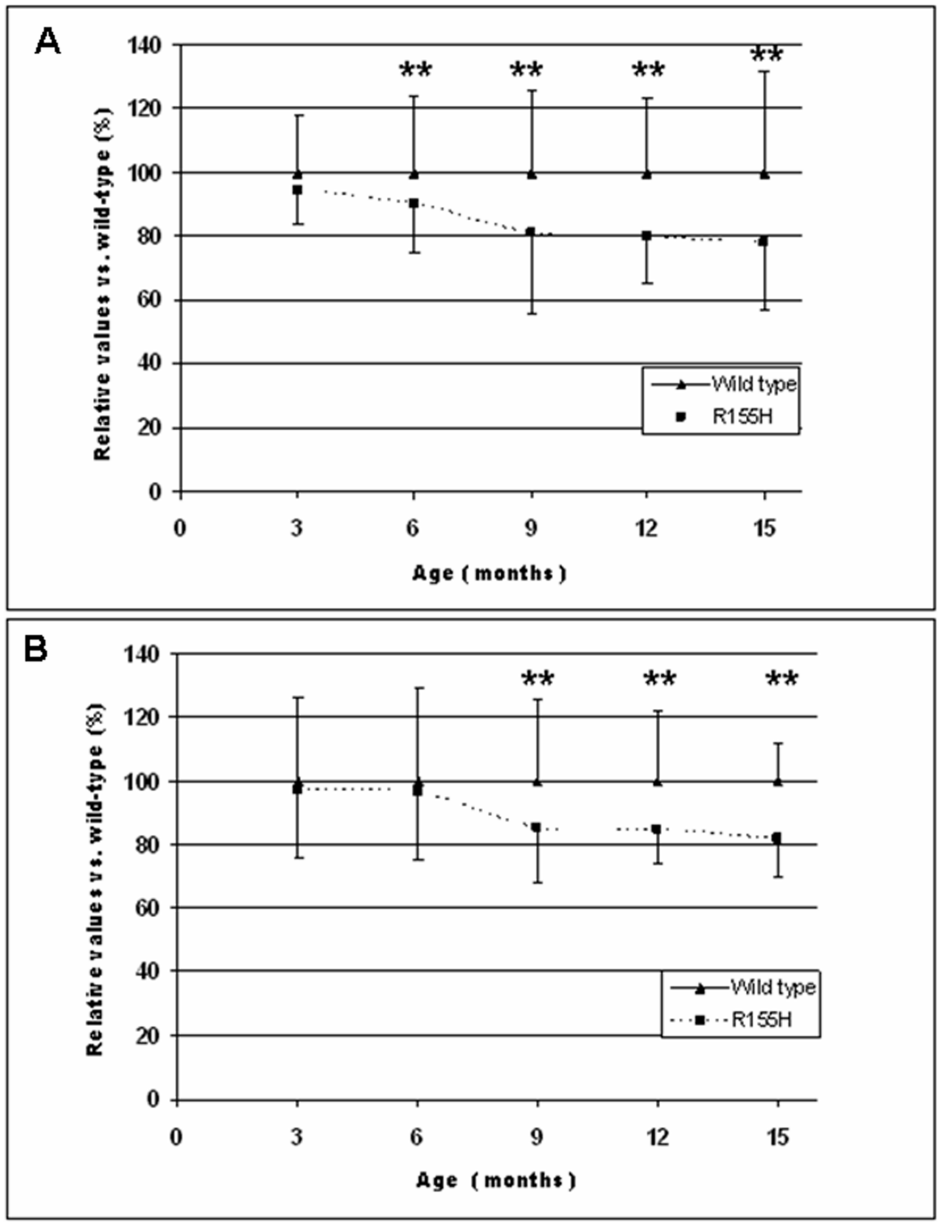
Progression of muscle weakness of the quadricep muscles in knock-in mice. (A) Decline of physical performance by Rotarod analysis in knock-in mice. (B) Progressive impairment of muscle strength measured by grip strength meter in knock-in mice. Ages of mice are shown in the X-axis and results are indicated in the Y-axis as relative values when compared to the wild-type mouse values. The following numbers of mice have been used to analyze physical performance test at different time points: 3 months (25 wild-type, 9 knock-in), 6 months (51 wild-type, 20 knock-in), 9 months (48 wild-type, 18 knock-in), 12 months (24 wild-type, 9 knock-in), and 15 months (20 wild-type, 8 knock-in). Note * * in the figure represents p value <0.05.

The Rotarod analysis in these mice showed a 5.5% decrease at the age of 3 months (p = 0.27), 9.7% decrease at 6 months (p = 0.05), 19.3% decrease at 9 months (p = 0.0072), 20.3% decrease at 12 months (p = 0.005), and 22.0% decrease at the age of 15 months (p = 0.04) in knock-in mice (**Figure 2A**). In the grip strength test, the knock-in mice demonstrated progressive muscle weakness in forelimbs. The decrease in muscle strength was consistent with the Rotarod analysis, showing a 2.5% decrease at 3 months (p = 0.75), 3.6% at 6 months (p = 0.60), 14.5% at 9 months (p = 0.028), 15.4% at 12 months (p = 0.05) and 17.9% at the age of 15 months (p = 0.05) (**Figure 2B**). There was no significant difference in mortality between KI and WT littermates when followed up to 15 months.

### Knock-in mice share similar histopathological features as patients

**Figure 3:**
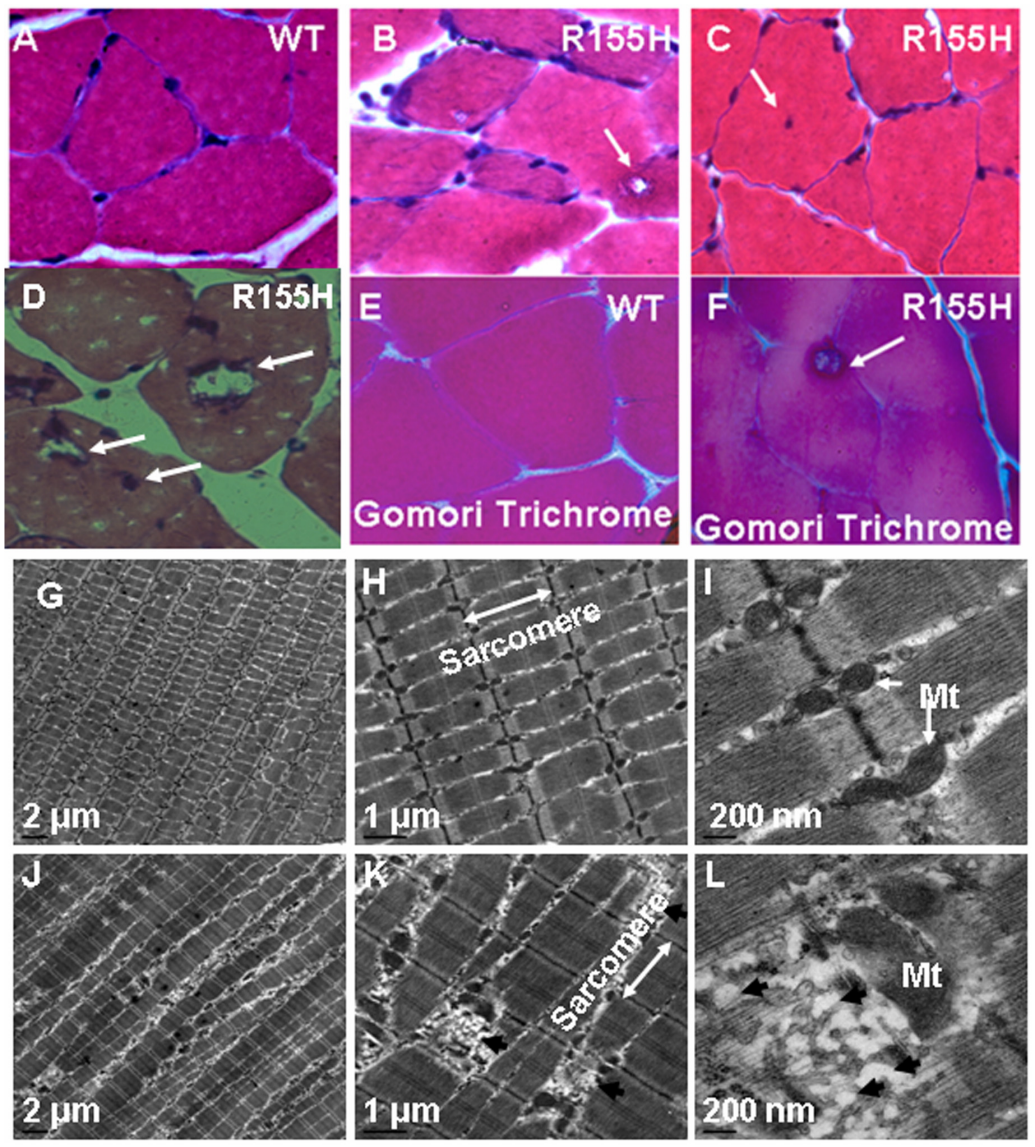
Immunohistochemical analysis in the wild-type and VCP^R155H/+^ knock-in mouse muscle. (A–C) Quadricep muscles from 9–10 month-old wild-type and VCP^R155H/+^ knock-in mice were analyzed by H&E staining. (B) An enlarged vacuole in the mutant tissue is shown by white arrows and (C) centrally located nuclei and rimmed vacuoles are revealed in the mutant mice shown by white arrows. (D) Quadriceps muscles from 15-month old VCP^R155H/+^ knock-in mice, centrally located nuclei shown by white arrows. (E–F) Modified Gomori Trichrome staining of muscle fibers from wild type and VCP^R155H/+^ knock-in mice. Magnification: 630×. (G–L) Electron microscopy analyses of the mouse quadricep muscles. Vacuolization and loss of myofilament organization are observed in quadriceps muscle from 10-month-old VCP^R155H/+^ knock-in mice (G–I), but not in wild-type mice (J–L). Sarcomeric direction is indicated by white double ended arrow (H,K). Swollen mitochondria are also observed in the mutant tissue (L). Black arrows in (K) and (L) indicate accumulation of vacuoles. Size bars are shown in the lower left corner of each image. Mt  =  mitochondria. Magnifications: E+H  = 900×, F+I  = 2,950×, G+J  = 11,500×. (N = 3 WT and 3 R155H animals).

Histological analyses of muscle tissues from human IBMPFD patients have revealed that patients' muscle fibers accumulate enlarged vacuoles as well as ubiquitin- and TDP-43-positive inclusion bodies [8]. To analyze if knock-in mice mimic human histopathology, we analyzed quadriceps tissues from 9–10 and 15-month old wild-type and knock-in mice by H&E staining, immunohistochemical and electron microscopy analyses. Structural analyses performed by H&E staining revealed that the quadriceps muscle from knock-in mice show variation in fiber size and accumulate enlarged rimmed vacuoles (**Figure 3A–C**). The analysis of 1,200 cells revealed that 2.8% of muscle cells from 9–10-month old and 15.4% of 15-month old knock-in mice were positive for vacuoles (**Table 1**). Vacuoles were not found in muscle tissues from 9–10-month old control mice, but 15-month old control muscle demonstrated vacuolization in 3.4% of cells. Additionally, 2.2% of muscle cells from 9–10-month old and 16.0% of 15-month old knock-in mice demonstrated centrally located nuclei by H&E staining (**Figure 3C–D**) compared to central nuclei in 7.7% of muscle cells from 15-month old control mice (p<0.05). Modified Gomori Trichrome staining revealed the presence of rimmed vacuoles in the R155H knock-in mouse when compared to wild-type controls (**Figure 3E–F**).

**Table 1:** Vacuoles, inclusion bodies and centrally located nuclei in 9–10 month and 15 month old wild-type and R155H knock-in mouse muscle.

Histological Methodology	Observed organelles	Results N = 3		Age (months)	Cells counted
		WT (%)	R155H (%)		WT/R155H
H&E	Vacuoles	0	2.75±0.432 p<0.05	9–10	1,200/1,200
TDP-43/Ub IHC	Inclusion bodies	0	3.96±0.331 p<0.05	9–10	1,386/1,386
H&E	Centrally located nuclei	0	2.16±0.144 p<0.05	9–10	1,200/1,200
H&E	Vacuoles	7.66±1.01	15.428±2.41 p<0.05	15	1,200/1,200
TDP-43/Ub IHC	Inclusion bodies	3.410±0.38	9.00±0.902 p<0.05	15	1,200/1,200
H&E	Centrally located nuclei	2.08±0.38	16.010±0.99 p<0.05	15	1,200/1,200

**H&E - Hematoxylin and Eosin; Ub – Ubiquitin; TDP-43 – TarDNA Binding Protein-43; IHC- Immunohistochemical staining; WT- Wild Type; R155H – Heterozygous knock in.**

Ultrastructural analyses of quadriceps muscle showed disorganized sarcomeres and vacuolization of muscle fibers in the knock-in mice as demonstrated in 12-month old mice (**Figure 3G–I**). Mutant muscle also showed abnormal swelling of mitochondria. The ultrastructural changes of the quadriceps from 9–10-month old mutant mice were milder when compared to older mice (**Figure 3J–L**).

### Characterization of muscle pathology in knock-in mice

**Figure 4:**
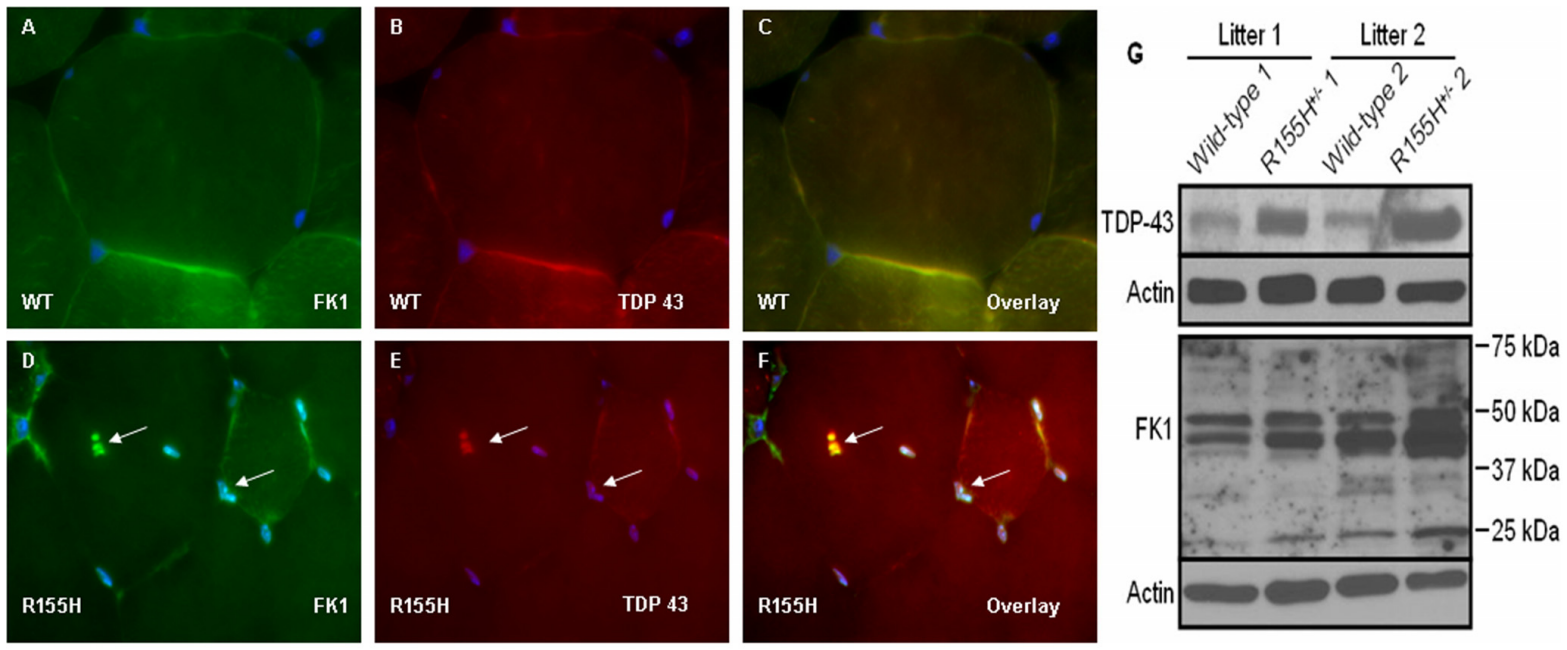
Immunohistochemical analysis and protein expression in the wild-type and VCP^R155H/+^ knock-in mouse muscle. (A–F) Immunohistochemical analysis of quadriceps muscles from 9–10 month old wild-type (A–C) and VCP^R155H/+^ knock-in mice (D–F) were stained with a ubiquitin-specific FK1 antibody (A,D) and a TDP-43-specific antibody (B,E). (C) shows the overlay of (A) and (B), and (F) is the overlay of (D) and (E). Ubiquitin- and TDP-43-positive, cytoplasmic inclusion body is shown by an arrow in (D–F). Nuclei were stained with DAPI. Magnification: 630×.). (G) Expression of TDP-43 and ubiquitinated proteins. Proteins were harvested from the quadriceps muscle of 2 littermates of wild-type and knock-in mice and analyzed by Western blotting using TDP-43 (upper panel) and ubiquitin/FK1 (lower panel) antibodies. Each membrane was re-probed with actin to confirm equal protein loading in each lane. Protein bands are indicated on the left and molecular weights of marker bands for the ubiquitin blot on the right. Genotypes are shown above. Wild-type and knock-in samples are from two litters (indicated above the figure) (N = 4 WT and 4 R155H animals).

To further characterize the muscle pathology, we stained fixed muscle sections with VCP-, ubiquitin- (FK1), and TDP-43-specific antibodies. Four percent (9–10-months old) and 9.0% (15-months old) of mutant cells had inclusion bodies that were positive for the TDP-43 and ubiquitin-specific antibodies, versus 2.1% in 15-month old control mice, (**Figure 4** **and** **Table 1**). The mutant knock-in muscle sections revealed strong expression of FK1 and TDP-43 (**Figure 4D–F**) as compared to the wild-type controls (**Figure 4A–C**). Western blotting analysis of two separate litter-matched wild-type and knock-in mice revealed that the mutant muscle had more TDP-43 and ubiquitinated (FK1 marker) proteins (**Figure 4G**). These inclusions were negative for the VCP antibody suggesting that mutant VCP does not accumulate in the mutant muscle cells.

### Autophagy and apoptosis is increased in knock-in mice muscle tissue

**Figure 5:**
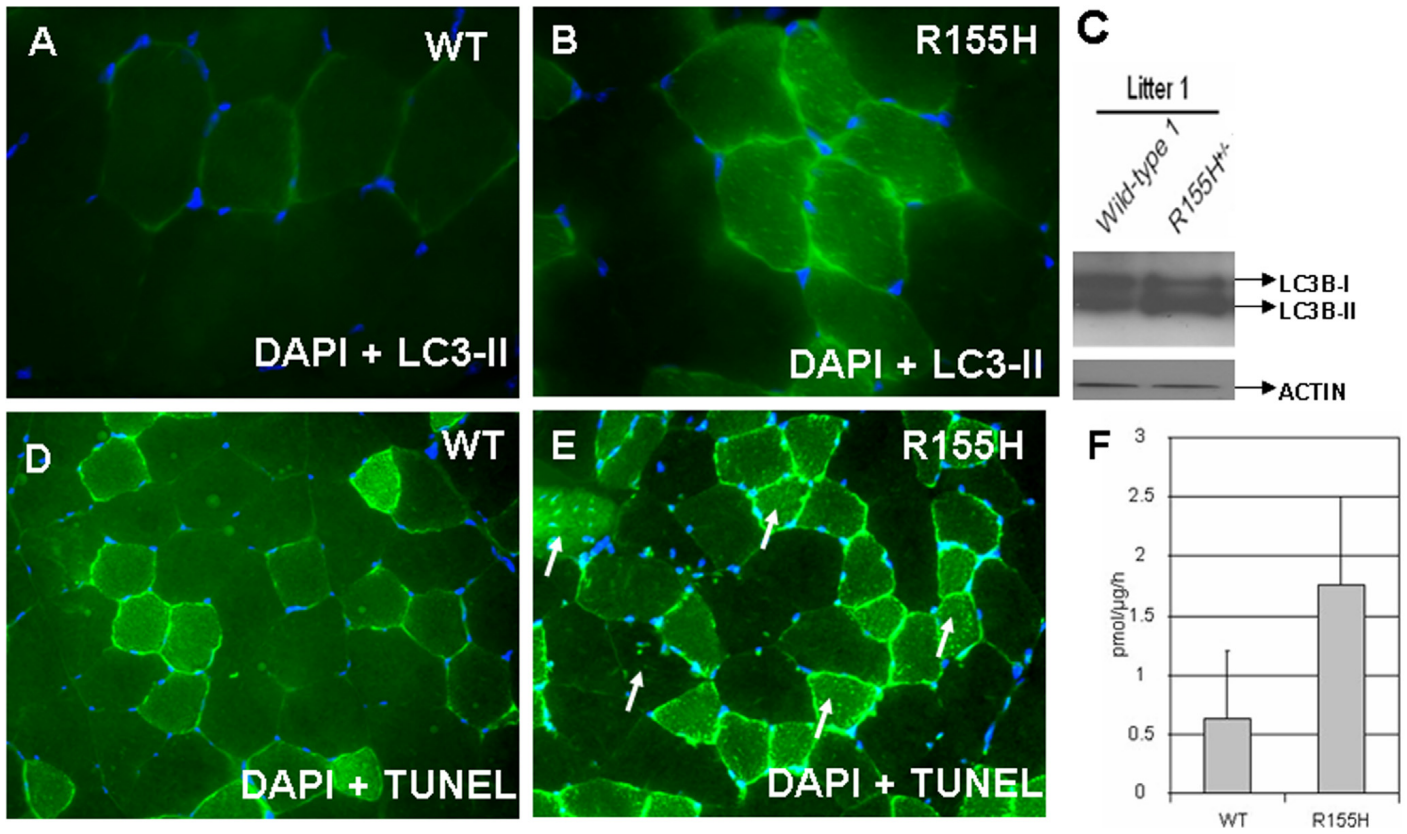
LC3-II staining, protein expression and apoptosis detection in the wild-type and VCP^R155H/+^ knock-in mouse quadriceps. (A–B) Quadricep muscles from 9–10 month old wild type and VCP^R155H/+^ knock-in mice were stained with an LC3-II-specific antibody. Nuclei were stained with DAPI (Magnification: 630×). (C) Protein expression of LC3-II is increased in knock in mice as compared with wild type litter mates. (D–E) DAPI and TUNEL staining of the quadriceps section from a wild-type and VCP^R155H/+^ knock-in mouse models (N = 3 WT and 3 R155H animals). Apoptotic nuclei of the mutant tissue are shown by white arrows. Magnification: 400×. (F) Caspase-3 activity was measured from the quadriceps muscle lysates of wild-type and VCP^R155H/+^ knock-in mice. Specific activities are shown in the Y-axis and genotypes in the X-axis (N = 4 WT and 4 R155H animals).

Autophagy is a process that degrades long-lived proteins and cytoplasmic components within vesicles which deliver the contents to the lysosome/vacuole for degradation. LC3 (ligh chain 3) serves as a marker for autophagy. There are three human autophagy forms of LC3B and LC3C). Upon activation of autophagy, the 18 kDa cytosolic LC3 (LC3B-I) undergoes proteolytic cleavage followed by a lipid modification and is converted to the 16 kDa membrane-bound form (LC3B-II), which is specifically localized to the autophagosomal membranes. The conversion from LC3B-I to LC3B-II is used as a sensitive marker for autophagy in cells. Using myoblasts obtained from patients with VCP associated IBM [9], [33] we observed accumulation of enlarged vacuoles as well as other several cellular dysfunctions in patients' myoblast cells versus normal control myoblasts. Western blotting analysis demonstrated that the protein lysates extracted from the mutant cells have significantly increased amount of LC3B-II when compared to the wild-type cell lines. Thus, to analyze if autophagic processes were also disrupted in the knock-in mice, we analyzed the expression of the LC3B-II protein. Muscle cells from the knock-in mice exhibited increased LC3B-II staining, which was concentrated in the vesicular organelles throughout the cytoplasm compared to WT (**Figure 5A,B**). Western blot analysis from knock-in VCP^R155H/+^ quadriceps tissue lysates demonstrated a significantly increased amount of LC3B-II when compared to the wild-type animals (**Figure 5C**).

Mutations in the VCP gene have been shown to trigger cell death with apoptotic features, whereas expression of wild-type protein has been suggested to have an anti-apoptotic effect [34], [35], [36]. These findings suggested that mutated VCP may also cause apoptosis in mouse muscle expressing the R155H disease mutation. To elucidate if this hypothesis holds true, we analyzed mouse quadriceps tissues for apoptosis by measuring caspase-3 activity and TUNEL staining. Mutant muscle showed 2.8-fold increase in caspase-3 activity when compared to the wild-type muscle (wild-type: 0.629 pmol/µg protein/hr; knock-in: 1.755 pmol/µg protein/hr) suggesting that mutant tissue exhibits significantly increased apoptosis (p = 0.007) (**Figure 5F**). These results were confirmed by TUNEL-staining of quadriceps sections, which showed increased number of apoptotic nuclei as compared with WT (wild-type; 6.9% and knock in: 28.9%, P<0.05) (**Figure 5D,E**). Some of the apoptotic nuclei were centrally located in the knock-in muscle.

### Bone pathology in VCP^R155H/+^ knock-in animals resembles Paget's Disease of Bone

**Figure 6:**
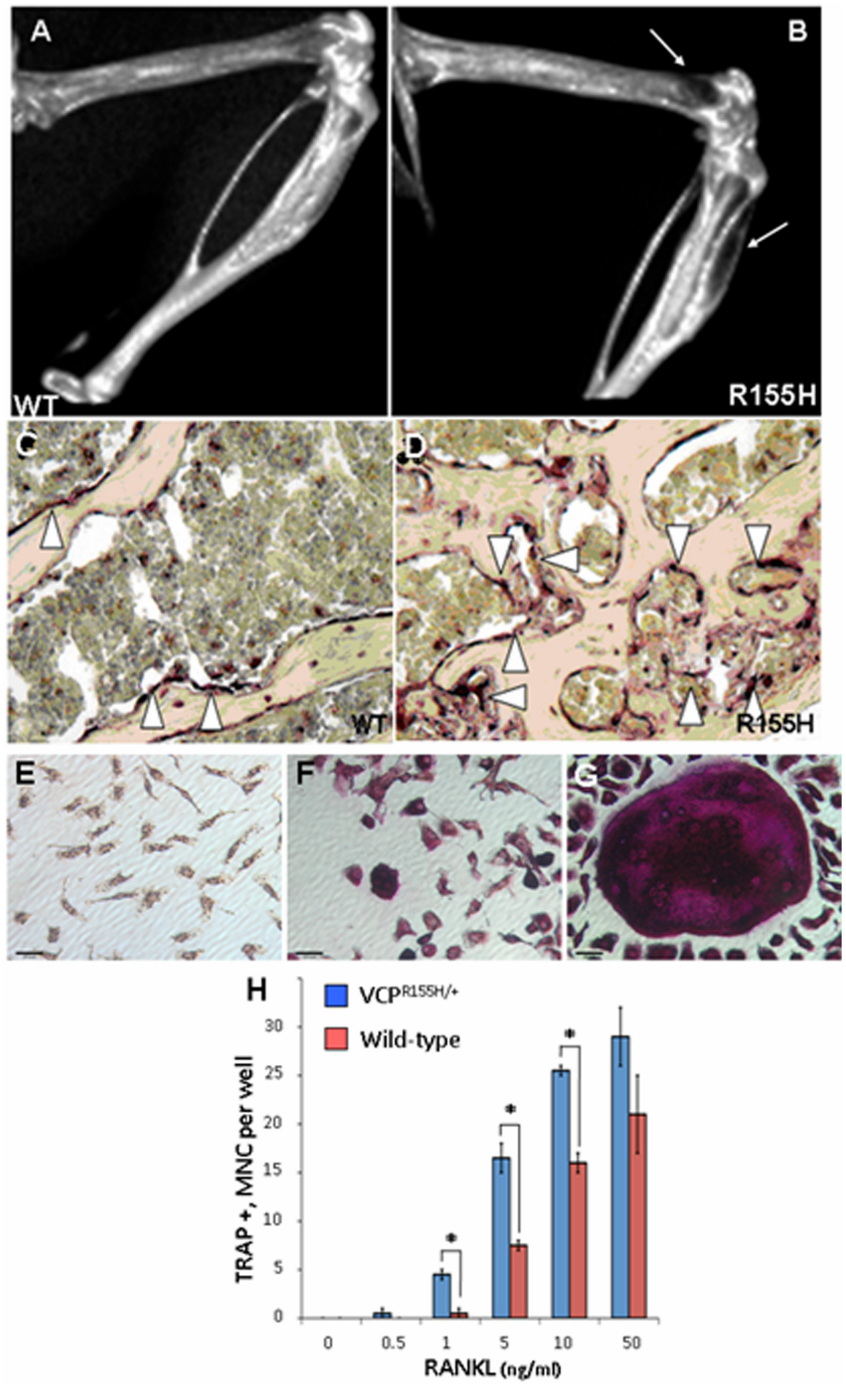
Bone micro CT imaging, histology and OCL formation of VCP^R155H/+^ and wild-type mice. (A–B) micro CT images showing sclerotic lesions at anterior tibia and posterior femur shown by white arrows in 15-month old knock-in mice. (C–D) Transverse sections of decalcified 6^th^ lumbar vertebra, white arrowheads indicate red colored TRAP-positive osteoclasts (Magnification 10×). (E–G) OCLs formed from non-adherent marrow cell cultures for WT or VCP^R155H/+^ cultured for 9 days in the presence of 10 ng/ml M-CSF and varying concentrations of RANKL. The cells were then fixed and stained for TRAP activity. Results represent TRAP positive cells containing ≥ three nuclei and are expressed as the mean ± SEM for duplicate cultures.*P<0.05 compared with results from wt cell cultures. MNC =  multi-nuclear cell. (G) Large TRAP positive multinuclear OC-like cell from knock-in bone marrow derived macrophages (BMDM). Differentiated in 100 ng/ml RANKL and 50 ng/ml M-CSF then fixed and TRAP stained on day 9 of differentiation. (H) Osteoclastogenesis cytokine sensitivity was determined with increasing concentrations of RANKL. TRAP-expressing multi-nucleated OCs was scored (N = 4 WT and 4 R155H animals).

To further characterize the bone pathology of knock-in mice we analyzed bone morphology by micro CT imaging. The micro CT images showed radiolucency of the proximal tibia and distal femurs in some knock-in mice (**Figure 6A,B**). The Bone Surface area/Bone Volume (BS/BV), Trabecular Space (Tb.Sp) was not different, but significantly decreased Trabecular Pattern Factor (Tb.P.F) and increased Cortical wall Thickness (C.Th) in knock-in mice was observed compared to WT mice (**Table 2** **)**.

**Table 2:** Bone Morphometry in WT and R155H KI mouse models.

Bone Morphometry		
Parameters	Wild Type	R155H KI
Bone Volume/Total Volume	0.68±0.17	0.7±0.12 p = 0.590
Bone Surface Area/Bone Volume	20.1±5.9	17.2±3.2 p = 0.062
Trabecular Thickness	0.107±0.03	0.12±0.02 p = 0.170
Trabecular Number	6.52±1.98	6.02±1.4 p = 0.360
Trabecular Space	0.07±0.11	0.053±0.031 p = 0.470
Trabecular Patter Factor	5.05±3.14	2.75±1.74 p = 0.007
Cortical Thickness	1.77±0.32	2.35±0.35 p = 0.0001

Significant differences were identified in the trabecular pattern factor and cortical wall thickness.

Histology of the tibiae and the centrum of the VCP^R155H/+^ mouse vertebra, when compared to wild-type, clearly showed excessive bone formation and increased numbers of osteoclasts. To further study osteoclast formation in our VCP^R155H/+^ mice, we performed TRAP (Tartarate Resistant Acid Phosphatase) staining of the tibia and 6th lumbar vertebra sections. A similar structure pattern and osteoclast distribution in VCP^R155H/+^ and wild-type without TRAP staining was noted (**Figure 6C–D**). TRAP staining of VCP^R155H/+^ tibia and centrum tissues demonstrated increased bone and osteoclasts as compared with wild-type at 10× (**Figure 6C–D**).

To study osteoclast formation in knock-in mice, bone marrow derived macrophages (BMDMs) and spleen-derived cells from wild-type and knock-in mice were cultured for 9 days in the presence of 10 ng/ml M-CSF and varying concentrations of RANKL (0, 0.5, 1, 5, 10 and 50 ng/ml). The cells were then fixed and stained for TRAP activity. Cultures of BMDM cells from knock-in mice formed significantly more osteoclasts (OCLs) in response to RANKL than wild-type (WT) BMDM cultures (**Figure 6E–G**), which is similar to bone marrow cultures from PDB patients. BMDM cultures from the R155H mice had significantly greater numbers of OCLs for 1, 5 and 10 ng/ml RANKL, but at 50 ng/ml the statistical significance was lost. Like OCLs formed in PDB patients BMDM cultures from the VCP^R155H/+^ mice contain a population of giant OCLs that have an increased number of nuclei per OCL compared to wild-type mice (**Figure 6G**). These giant cells were not seen in any wild-type culture during the course of these experiments and the giant OCLs were a sub-population of OCLs in the mutant BMDM and spleen cultures, where most multi-nuclear TRAP positive OCLs were of similar size as those in the wild-type cultures. The increase in TRAP positive multinucleated cells observed in knock-in mice suggested increased osteoclastogenesis associated with Paget-like disease of bone (**Figure 6H**).

### Brain tissue analysis

**Figure 7:**
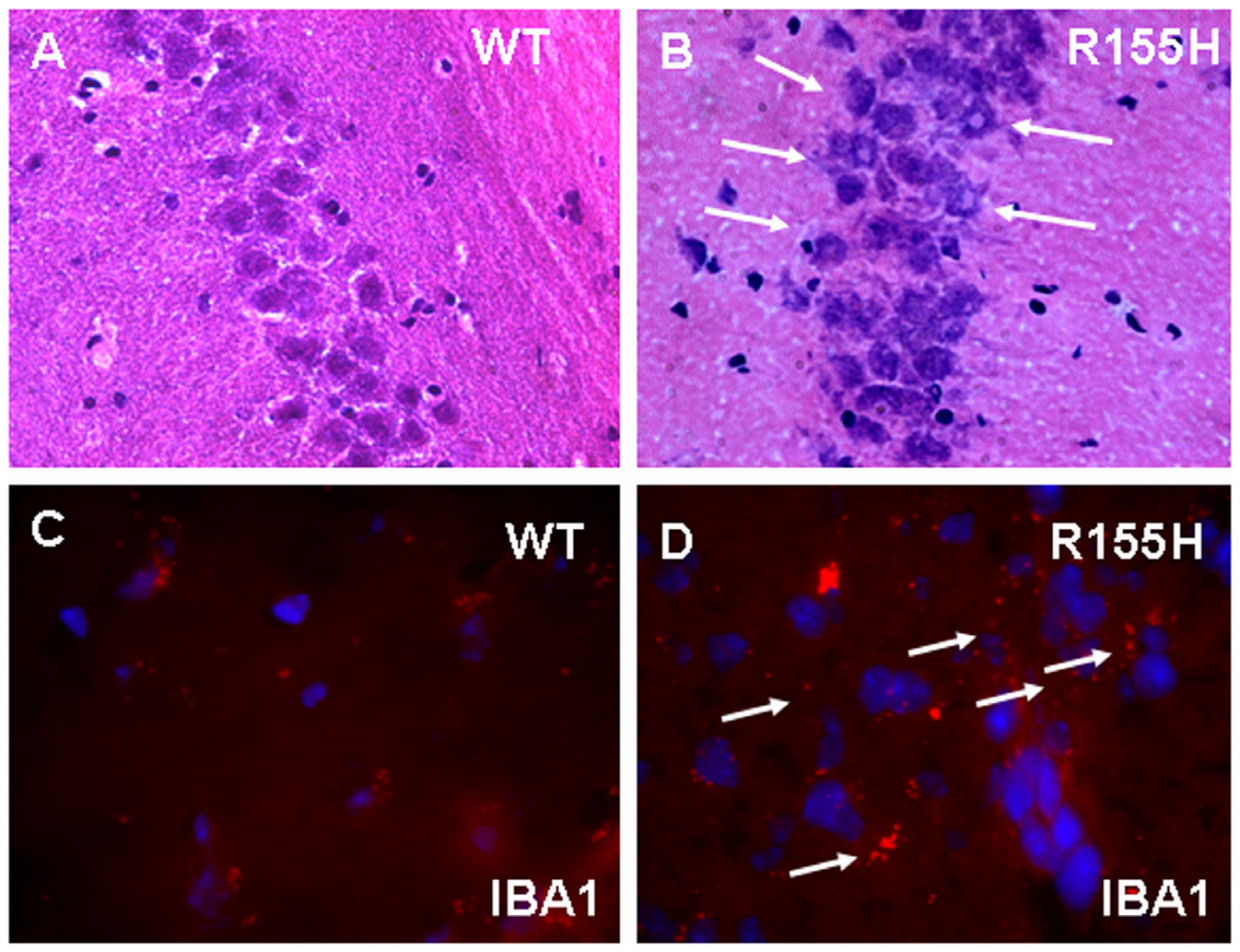
Immunohistochemical brain staining of the wild-type and R155H knock-in mouse. (A–B) H&E staining of 15 month old wild type and knock-in mouse of hippocampus and neuronal injury shown by white arrows (Magnification 400×). (C–D) IBA1 staining of frontal cortex from wild-type and R155H knock-in mice shown by white arrows Magnification: 630× (N = 3 WT and 3 R155H animals).

**Figure 8:**
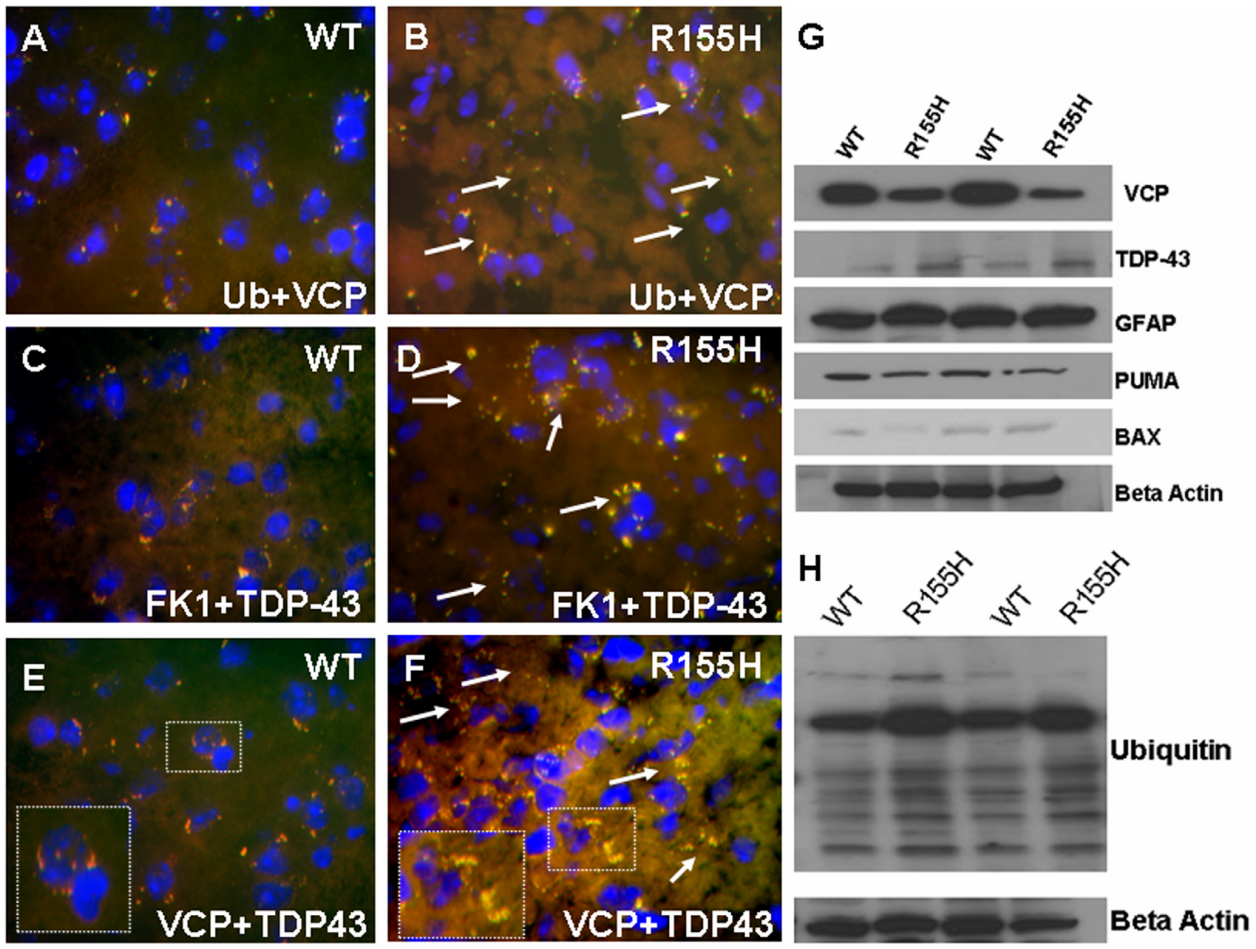
Immunohistochemical staining and protein expression of the wild-type and R155H knock-in mouse brain. (A–B) Co-localization of VCP- and ubiquitin-specific antibodies in 15-month old wild type and R155H knock-in mice. (C–D) Co-localization of FK1- and TDP-43-specific antibodies in wild type and R155H knock-in mice from frontal cortex. Inclusion bodies are shown by white arrows. Nuclei were stained with DAPI (blue). Magnification: 630× (N = 3 WT and 3 R155H animals). (E–F) Co-localization of VCP- and TDP-43-specific antibodies of wild type and R155H knock-in mice from frontal cortex. Inclusion bodies are positive for VCP and TDP-43 antibodies are shown by white arrows and inset. Nuclei were stained with DAPI. (G–H) Protein expression of VCP, TDP-43, GFAP, PUMA, BAX and ubiquitin have been analyzed by Western immunoblot from WT and R155H KI mutant whole brain extracts (N = 4 WT and 4 R155H animals). Beta actin was used as loading controls for these Western blot analyses.

Similar inclusion pathology has been reported in patients' brain, including neocortex, as well as in limbic and subcortical nuclei. Histological analyses of the brain tissues from 9–10-month and 15-month old mutant mice did not show any overt histological signs of degeneration in H&E staining (**Figure 7A,B**). Ionized Calcium-binding adaptor molecule 1 is expressed selectively in microglia and is upregulated during activation of cells. Staining of the frontal cortex showed increased IBA1 expression in the mutant versus wild type mice (**Figure 7C,D**). However, the immunohistochemical staining of frontal cortex from 15-month old knock-in mice demonstrated increased TDP-43 and ubiquitin-positive inclusions. In contrast to muscle samples, these cortical inclusions were also positive for the VCP antibody. Staining with Ubiquitin and VCP demonstrated cytoplasmic inclusions in the frontal cortex in the mutant mice as compared with the WT (**Figure 8A,B**). Similar findings were observed in the mutant and WT mice with FK1- and TDP-43-specific staining (**Figure 8C,D**). The TDP-43 inclusions showed nuclear clearance and cytoplasmic accumulation in the knock-in mouse brains whereas the TDP-43 inclusions showed nuclear localization in the cortex of control knock-in mice (**Figure 8E,F**). These results were confirmed by western blot analyses of VCP, TDP-43 (**Figure 8G,H**). GFAP (glial fibrillary acidic protein) and pro-apoptotic markers including p53 upregulated modulator of apoptosis (PUMA) and Bcl-2–associated X protein (BAX) were analyzed by western blot analysis and no significant differences were found in the R155H versus WT mice (**Figure 8G**).

### 6- and 15-month old knock-in mice do not show impaired short term memory, but show seizures

To analyze if knock-in mice exhibit dementia, we performed a novel object recognition test with the 6- and 15-month old mice. Values closer to 50% suggest that the mice show no object preference, whereas values closer to 100% suggest that the mice prefer the novel object. No significant difference in novel object recognition test was observed between 6-month old wild-type and knock-in mice. The memory index of the 15-month old heterozygous mice was 67.8%, with the wild-type mice scoring 55.2% (p = 0.09) suggesting that the short term memory of the 15-month old knock-in mice is not impaired.

Unexpectedly, we observed complex seizures in the heterozygous mice. These seizures started with partial, limbic behavior, followed by tonic-clonic seizures (clonic wild running and somersaulting was also observed) and ended by a typical post-ictal quiescent phase including repetitive behavior (grooming). Four heterozygous mice out of 27 (14.8%) developed seizures at the age of 9 months, and another three out of an additional 30 (10%) at the age of 12 months. The seizures were precipitated by moving or tapping on the cage of these mice, but they did not appear to shorten the lifespan of the mice. Seizures did not shorten the life span of the mice. Seizures were not observed in wild-type age-matched littermates up to the age of 15 months (https://webfiles.uci.edu/xythoswfs/webui/_xy-6368126_1-t_VqHhatlP).

## Discussion

VCP is involved in a number of cellular functions, most of which are related to ubiquitin-proteasome dependent proteolysis [24], [37]–[40]. It is highly conserved in evolution suggesting an essential role for normal cellular functions in both unicellular (yeast) and multi-cellular organisms [41]–[43]. The finding that inhibition of VCP expression promotes apoptosis [44] suggests that intact VCP is indispensable for normal development and cell survival. Our recent studies of myoblasts associated from subjects with VCP mutations demonstrated accumulation of enlarged vacuoles [9]. Despite intense investigations, the disease mechanisms underlying IBMPFD are yet to be clarified. Our knock-in mice exhibit muscle weakness, vacuoles and inclusions in the muscle fibers, Paget like bone changes on microCT and histology and typical brain pathology of IBMPFD. Collectively, these findings suggest that our mouse model provides an important experimental platform for probing the underlying mechanisms responsible for this unique disease. The following discussion addresses each of these key findings.

### VCP^R155H/+^ myopathy and its correlation to that in humans

To be able to elucidate tissue and cellular pathology of IBMPFD, we have generated a knock-in mouse model that expresses the most common VCP disease mutation R155H at endogenous level. By creating a mouse model that mirrors the human disease and by studying the muscle, brain, bone and other organs in the mice, we hope to be able to understand the enormous clinical variability in IBMPFD patients. Furthermore, our current mice provide us with the potential of breeding new mouse models to better understand the increased risk of FTD observed in those with both an APOE4 allele and mutant VCP phenotype [38].

Previous animal models for IBMPFD include the knock-out mouse model reported by Muller et al (2007) [30]. Mice with homozygous deletion of VCP did not survive to birth, but died at a peri-implantation stage, whereas hemizygote mice were indistinguishable from their wild-type littermates. When hemizygote mice were mated, no homozygous mice were ever seen endorsing an important role for VCP in normal development of mouse embryos. Transgenic mice overexpressing R155H VCP under a muscle-specific promoter, reported by Weihl et al (2006) [31], demonstrated muscle weakness, and slit-like vacuoles. Additionally, Custer et al. have generated transgenic mice expressing VCP/p97 with the R155H and A232E mutations shown to develop pathology of the muscle, bone, and brain exhitibing similar characteristics seen in humans with IBMPFD [32].

The VCP^R155H/+^ allele in the knock-in mouse model that we have created expresses the mutant allele at physiological levels as seen in the patients and thus overcomes the problems of over expression. Our knock-in mice demonstrates several phenotypic and histological changes, all of which are typical of human IBMPFD patients, who develop muscle weakness at the mean age of 42 years. Progressive deterioration of muscle strength of knock-in mice mirrors the development of late onset muscle weakness reported in patients [1], [3], [45] and transgenic mice expressing R155H VCP [31].

Histological analyses of muscle from our knock-in mouse model of IBMPFD reveal vacuolization and central nucleation TDP-43-and ubiquitin-positive sarcoplasmic inclusions [8] thus, closely matching the histopathology of human IBMPFD patient muscle. Electron microscopic studies of the mutant muscle showed disorganization and vacuolization of the sarcomeres [46]–[48] and significant swelling of mitochondria associated with increased apoptosis, which is not only seen in our knock-in mice and human IBMPFD, but also in yeast [49] supporting the high evolutionary conservation of VCP from yeast to human (70%) [41] and suggesting that energy metabolism of affected muscle may be disturbed.

### Bone remodeling in the VCP^R155H/+^ mouse model: correlation to bone pathology in IBMPFD patients

PDB is characterized by abnormal bone remodeling, osteolytic lesions, bone deformities and pathologic fractures (in severe cases). An initial surge of osteoclastic activity leads to focalized resorption of bone, followed shortly thereafter, by osteoblast hyperactivity. The osteoblasts are normal morphologically, but deposit new bone in a disordered manner resulting in Pagetic lesions. The new disorganized bone is of poor quality leading to bowing and occasional fractures. In the more advanced stages of Paget disease, rapid bone formation predominates. The lesions become sclerotic, bone marrow is replaced with vascular and fibrous tissue and increase in bone thickness. Using histochemical stains and microCT imaging, we compared the bone findings of our knock-in mice to the typical findings in human Paget's disease. The microCT images of knock-in mice show radiolucency of the distal femurs and proximal tibias which is consistent with bone morphometric analysis of our knock-in mice. Osteoclasts in Paget-like lesions are increased in both number and size indicated by TRAP immunostaining. In vitro studies of bone marrow derived macrophages from knock-in mice femurs and tibia revealed hyper responsiveness to RANKL. Finding that knock-in mutant mice can generate giant osteoclasts is an important result, since this cellular phenotype resembles the human Pagetic osteoclast phenotype. The localized lesions seen in the mice also resemble the patchy distribution of Pagetic lesions in patients. The IBMPFD mouse promises to be a good model for human Paget disease of the bone for future therapeutic studies.

Our studies suggest that our generated knock-in mouse model for IBMPFD represents an excellent model for the human disease. Our mice express the R155H mutant VCP gene at an endogenous level compared to previous models which overexpresses the mutant allele. Our knock-in mice provide an opportunity to study the molecular pathways regulating the myriad aspects of VCP disease and thus, may aid in the development of novel strategies for treatments of diseases with similar pathogenesis.

Our recent studies in IBMPFD patient myoblasts exhibit increased apoptosis when analyzed by caspase-3 assays and TUNEL staining as well as increased autophagy [9]. We replicated our studies in the knock-in mouse muscle, and observed similar findings. Autophagy is a process that functions as a stress response that is upregulated by starvation, oxidative stress, or other harmful conditions. It plays a role in programmed cell death and possesses important housekeeping and quality control functions that contribute to health and longevity (for review, see [50]–[53]. In addition, impaired autophagic degradation contributes to the pathogenesis of several human diseases including muscle diseases and lysosomal storage disorders. Danon disease is an example of a human myopathy that is characterized by accumulation of autophagic vacuoles in the heart and skeletal muscle [54]. A mouse model for Danon disease exhibits similar histological and phenotypical features to human patients showing massive accumulation of autophagic vacuoles in several tissues, including skeletal muscle and heart [55]. Vacuoles are seen in the muscle of the heterozygotes and were very prominent in the cardiac muscle in the homozygotes. Mitochondria are a main target for autophagic degradation in muscle diseases resulting in reduced mitochondrial function, which leads to defective energy metabolism and reduced contractility [56]. Therefore, increased autophagosome formation as exhibited by LC3-II accumulation, as well as muscle weakness seen in both our VCP^R155H/+^ knock-in mice and IBMPFD patients, is consistent with other myopathic conditions suggesting a common pathogenesis. Both knock-in mice and IBMPFD patient histology indicate not only induction of autophagy, but also disruption of the autophagic maturation process. The current hypothesis is that immature autophagosomes accumulate in tissues secondary to impaired lysosome-autophagosome fusion further contributing to tissue pathology.

### Brain histopathology in the VCP^R155H/+^ mouse model

Frontotemporal dementia develops in 27% of human patients at the mean age of 57 years [2]. Although no short term memory deficiencies were observed in knock-in mice, the development of brain histopathology was similar to that of human patients. We observed accumulation of TDP-43-, ubiquitin-, and VCP-positive inclusions in the 15-month old knock-in brain, but not in 10-month old brain, mimicking the delayed development of human brain pathology. Immunohistochemical staining of frontal cortex and hippocampus showed increased nuclear clearance and cytoplasmic accumulation of TDP-43 inclusions in the knock-in mouse brains similar to the pathology in humans with VCP disease, in contrast to the nuclear localization in the cortex and hippocampus of control mice. In addition, inclusions stain positive for TDP-43, VCP and Ubiquitin [57]. The ionized calcium binding adaptor protein 1 (IBA1) is thought to play a role in regulating the function of microglia and considered to be specific biomarker. Microglial primary function involves maintenance of normal tissue brain homeostasis and production of variety of neurotransmitters [58]. Higher levels of IBA 1 expression was observed in frontal cortex of knock-in mouse. Hence homeostasis of brain and neurotransmission may be altered. Glial fibrillary Acidic protein (GFAP) is astrocyte marker and reveals morphological changes during cell injury or adverse conditions [59]. The immunohistochemical staining and western immunoblot analysis have shown no changes in both knock- in mouse and control. The morphological changes in astrocytes and astrogliosis are not involved in TDP pathology of knock-in mouse. The apoptotic pathway has not been induced in brain as there is no alteration in pro-apoptotic markers (PUMA, BAX).

Interestingly, we observed spontaneous seizures in 12.3% knock-in mice. Since seizures are not reported in human IBMPFD patients, these findings combined with the observation that VCP is abundantly expressed in hippocampus [60] propose an important role for VCP in the hippocampal region. We observed neuronal injury in the hippocampus CA3 region (**Figure 7 A,B**) of knock-in mouse. Although seizures are not seen in VCP disease, they are a feature of early onset dementia and frontotemporal dementia [61]. As we study additional families and expand the phenotype of this multifaceted disease, we predict that patients/families will be identified in which seizures are an associated manifestation.

## Materials and Methods

### Ethics Statement

All experiments were done with the approval of the Institutional Animal Care and Use Committee (IACUC) of the University of California, Irvine (IACUC Protocol #2007–2716), and in accordance with the guidelines established by the NIH. Animals were housed in the animal facility of the University of California, Irvine, and maintained under constant temperature (22°C) and humidity with a controlled 12:12-h light-dark cycle. Mice had free access to mouse chow and 0.9% NaCl drinking water. Animal welfare including objective measures such as physiological and behavioral indicators were monitored and recorded four times a week. To ameliorate any suffering of animals observed throughout these experimental studies, mice were euthanized by CO_2_ inhalation.

### Generation of the VCP^R155H/+^ knock-in mouse model for IBMPFD

Mouse genomic VCP fragment with 7.9 kb of upstream homology sequence and 2.1 kb of downstream homology sequence was subcloned into a targeting vector. Site-directed mutagenesis using Quick-Change XL Site-Directed Mutagenesis Kit (Stratagene, La Jolla, CA) was used to introduce the R to H mutation at amino acid position 155. The knock-in mouse model with the R155H VCP mutation was generated at the inGenious Targeting Laboratory, Inc. (Stony Brook, NY). 10 µg of the targeting vector was linearized by NotI (New England BioLabs Inc., Ipswich, MA) and then transfected by electroporation into 129/SvEv embryonic stem (ES) cells. After the selection in the G418 antibiotic (Gibco, Carlsbad, CA), surviving clones were expanded for Southern blotting analysis, and recombinant ES cells were injected into 129/SvEv blastocysts, which were transferred to the uteri of pseudopregnant females. Resulting chimeras were mated to 129/SvEv mice to produce F1 generation of mutant mice. Before experiments, generated heterozygous VCP^R155H/+^ knock-in mice were back-crossed at least six times with C57BL/6JEiJ. Therefore, more that 98% of the genome is expected to originate from C57BL/6JEiJ. Littermates were used in every experiment.

### Validation of the VCP^R155H/+^ knock-in mice

For PCR genotyping, DNA was extracted from mouse tail samples using a DirectPCR Lysis Reagent (Viagen Biotech Inc., Los Angeles, CA) following the manufacturer's guidelines. Tail DNA samples were subjected to PCR using the following primers: Forward: 5′-gcc tct ctg aag gat aat gtg g-3′; Reverse: 5′-atg gga ttg ggt tct ttc aa-3′. PCR products were digested with MspI (New England BioLabs, Inc.) and the results were analyzed by agarose gel electrophoresis.

The expression of the mutant allele was confirmed by RT-PCR using the following primers in the PCR reactions: Forward: 5′-cac ggt gtt gct aaa agg aaa gaa aag; Reverse: 3′-ctg aag aat ctc caa acg tcc tgt agc, after the RT reactions with the Reverse primer. Amplified products were digested with MspI (New England BioLabs Inc., Ipswich, MA) and the results were analyzed by agarose gel electrophoresis. Total mRNA was isolated from quadriceps of mice (4 from knock-in and 4 from wild type) using TRIzol reagent (Invitrogen)) followed by the RNeasy Mini Kit with on-column DNase treatment. cDNA was prepared from total RNA (500 ng per 20 ml reaction) with the RT2 First Strand Kit (SA Biosciences, Frederick, MD), and 50 ng of cDNA in RT2 qPCR Master Mix (SA Biosciences) was applied to each well of the 384-well array, according to the manufacturer's instructions. Above primer sequences were used for both the RT-PCR and qRT-PCR. After running the Q-RT-PCR on Roche Light Cycler 480 instrument, the results were analyzed by using SA Biosciences online software (http://www.sabiosciences.com/pcr/arrayanalysis.php).

### Measurements of weight, motor coordination and muscle strength

To follow the development of body mass, the weight of every knock-in and wild-type mouse was measured weekly. Motor coordination and fatigue were assessed by a rotarod (Med Associates Inc., St. Albans, VT) accelerating speed analysis. The rotarod test was performed for all existing mice at the age of 3 months (25 wild-type, 9 knock-in), 6 months (51 wild-type, 20 knock-in), 9 months (48 wild-type, 18 knock-in), 12 months (24 wild-type, 9 knock-in), and 15 months (20 wild-type, 8 knock-in). Mice were placed on the rotarod, which accelerates from 4 to 40 rpm in 5 minutes. The results were recorded as a mouse dropped down from the rotarod for the first time. Mice went through three trials with 45- to 60-minute inter-trial intervals on each of two consecutive days. Data from the previous two day trial were used to set the baseline. Statistical analyses were performed by student's t-test.

Muscle strength of the forelimbs of mice was measured by a Grip Strength Test using a Grip Strength Meter apparatus (TSE Systems Gmbh, Hamburg, Germany) for the same mice as in the Rotarod test. Mice were held from the tip of the tail above the grid and gently lowered down until the front paws grasped the grid. Hind limbs were kept free from contact with the grid. The animal was brought to an almost horizontal position and pulled back gently but steadily until the grip was released. The maximal force achieved by the animal was recorded. Each animal underwent 5 testings.

### Novel object recognition test

Short term memory of 6-month old mice (6 wild type and 6 knock-in) and 18-month old mice (5 wild-type and 5 knock-in) was analyzed by novel object recognition test. In phase I (habituation), mice were habituated for three consecutive days for 20 min each day in their cages. During this habituation phase, no objects were introduced. On day 4, mice were familiarized with identical objects in their clean testing cages for 15 min. These objects were not used in the ensuing sessions. The novel object recognition test was performed the following week.

In phase II (object recognition test), mice were placed in separate cages, each containing two identical objects. Mice were allowed to explore the objects for 5 min, after which the mice were returned to their own cages for a 5-min retention period. Thereafter, mice were returned to their new testing cage. Upon return, one of the objects had been replaced with a new object. The mice were then allowed to explore for 3 min. All testing activities were videotaped and following well-documented procedures specifically designed for object memory tests [62].

The scoring of data was done by reviewing the recorded tapes by measuring the time mice spent with familiar and novel objects. The timing did not begin before until mice moved from their original position. Additionally, exploring was scored if the mouse was within one inch of the object. Activities such as chewing, grooming, or standing farther than one inch from the object were not scored. The memory index (MI) was determined using the formula MI  =  Tn ×100/(Tn + Tf), in which Tn  =  time spent with the novel object, and Tf  =  time spent with the familiar object.

### Histological analyses of muscle tissue

The histopathology of knock-in mice was analyzed by hematoxylin and eosin (H&E) staining, immunohistochemistry and electron microscopy. 9–10 and 15-month old mice (3 wild-type and 3 knock-in) were euthanized by cervical dislocation, dissected quadriceps and brain tissues were mounted in a cryo-sectioning mounting media (Electron Microscopy Sciences, Hatfield, PA) and stored at −80°C before sectioning at 5–10 µm. For H&E staining, cryo-sections were fixed in 10% formalin for 30 min and washed 3 times with PBS. Thereafter, sections were incubated in hematoxylin (Poly Scientific, Bayshore, NY)/glacial acetic acid (Sigma-Aldrich, St. Louis, MO)-solution for 3 min, rinsed with deionized water for 5 min, dipped 8–12 times into acid ethanol, rinsed 2 times for 1 min with tap water, and once with deionized water for 2 min. Thereafter, slides were stained with eosin (Poly Scientific) for 30–45 sec, incubated in 95% ethanol 3 times for 5 min, 3 times in 100% ethanol for 5 min, and finally 3 times in xylene for 15 min. Slides were mounted with Permount (Fisher Scientific, Pittsburgh, PA) and results were analyzed by a light microscope (Carl Zeiss, Thornwood, NY) using an AxioVision image capture system (Carl Zeiss).

For immunohistochemical analyses of mouse tissues, fixed sections were blocked with 5% fetal bovine serum/PBS solution for 30 min. The sections were immunostained with TDP-43 (Abcam, Cambridge, MA), FK1 (BIOMOL), VCP (Affinity BioReagents, Golden, CO), LC3 (Novus Biologicals, Littleton, CO), IBA1 (Ionized calcium binding adaptor molecule 1,WAKO, Richmond, VA), GFAP (Glial fibrillary acidic protein, DAKO, Denmark), BAX (Cell Signaling, Danvers, MA), and PUMA (Cell Signaling, Danvers, MA) antibodies overnight at 4°C, and washed 3 times with PBS. Fluorescein-conjugated secondary antibodies (Sigma-Aldrich, St. Louis, MO) were incubated for 1 hour at room temperature. Sections were washed three times with PBS before mounting in DAPI-containing mounting media (Vector Laboratories, Inc., Burlingame, CA). Modified Trichrome Gomori staining was performed with Gomori Trichrome Stain on quadriceps tissue sections using routine methods. Results were analyzed by a light microscope (Carl Zeiss) using an AxioVision image capture system (Carl Zeiss).

For electron microscopic studies, quadriceps muscles from three 9–10 and 12-month old knock-in and wild-type mice were fixed in 4% paraformaldehyde/0.1% glutaraldehyde/0.1 M PB. Then tissue samples were fixed in 1% glutaraldehyde overnight and in 1% Osmium for 1 h at 4°C. Samples were embedded in Eponate 12 resinin at 65°C for 24–36 h and serially dehydrated in ethanol. Ultra-thin (60∼80 nm) sections were cut with a diamond knife. Sections were stained in 1% uranyl acetate for 30 min, followed by lead citrate staining for 7–10 min. Sections were examined by the Philips CM10 transmission electron microscope (Amsterdam, The Netherlands), and electron micrographs were taken with a Gatan UltraScan US1000 digital camera (Philips).

### Protein expression analyses by Western blotting

To determine the expression levels of proteins in the mouse muscle, quadriceps muscle tissues were subjected to Western blotting. Muscle samples from 9–10-month-old wild-type and knock-in mice (4 wild-type and 4 knock-in mice from four litters, of which only 2 litter sets are shown in this manuscript) were harvested and lysed by homogenization and sonication in a RIPA-buffer (50 mM Tris pH 8.0, 150 mM NaCl, 1% igepal, 0.5% deoxycholic acid, 0.1% SDS; all from Sigma-Aldrich) supplemented with protease inhibitors (Halt Protease Inhibitor Cocktail; Pierce, Rockford, IL). Protein concentrations were determined using the Bio-Rad Protein Assay kit (Bio-Rad, Hercules, CA) according to the manufacturer's protocols. Equal amount of proteins were separated on SDS-PAGE gels, and the expression levels of proteins were analyzed by Western blotting using TDP-43 (Abcam, Cambridge, MA) and FK1-specific antibodies (BIOMOL Research Labs, Plymouth Meeting, PA). Equal protein loading was confirmed by a Beta-Actin antibody (Santa Cruz Biotechnology, Santa Cruz, CA) staining.

### Apoptosis

Apoptosis in mouse tissue samples was analyzed by the DeadEnd Fluorometric TUNEL System (Promega, Madison, WI) and by the Colorimetric CaspACE-3 Assay System (Promega). For TUNEL staining, muscle cryo-sections from two 10-month old VCP^R155H/+^ knock-in mice and two wild-type littermates were generated as described above. Sections were fixed with 4% paraformaldehyde, washed with PBS, permeabilized with 0.2% Triton X-100 (Sigma-Aldrich, St. Louis, MO) solution in PBS, rinsed with PBS, equilibrated with equilibration buffer, and labeled with nucleotide mix/rTdT enzyme solution. After washing with 2× SSC, cells were washed with PBS and mounted in Vectashield with DAPI. The results were analyzed by immunofluorescence microscopy (Carl Zeiss). For caspase-3 assay, muscle proteins were extracted with cell lysis buffer from the same mice as described above. To complete the lysis, samples were frozen and thawed three times after homogenization, followed by centrifugation. Supernatants were transferred into a fresh tube and protein concentrations were determined using the Bio-Rad Protein Assay kit (Bio-Rad, Hercules, CA). Equal amount of proteins were analyzed in 96-well plates in triplicate, in the presence of 2% DMSO, 100 mM DTT, and 0.2 mM DEVD-pNA substrate. Reaction mixtures were incubated at 37°C for 4 h and the results were obtained by measuring the absorbance at 405 nm. Standard curve was generated following the manufacturer's instructions.

### Bone histomorphometry

The mice were examined with an Inveon microCT (Siemen's Inc.) in vivo under mild anesthesia (2.5% isoflurane). The mice were scanned with a large area CT camera that has a 30–40 micron high resolution, low noise, 14-bit x-ray imaging detector with 4096×4096 pixels. (usable field of view for this configuration is 10 cm ×10 cm). The high performance 64-bit workstation controls the Inveon multimodality scanners and was also used for reconstruction of image data. The reconstructed microCT images were analyzed and 3D trabecular structural parameters were determined using the Inveon Multimodality 3D Visualization software. The Bone volume/Total volume (BV/TV), Bone surface area/Bone volume (BS/BV), Trabecular Thickness (Tb.Th), Trabecular Space(Tb.Sp), Trabecular Number (Tb.N) and Trabecular Pattern Factor (Tb PF) were analyzed from 4-wt and 4-mutant femurs (right and left femurs combined) in 15-month old mice [63], [64].

To elucidate osteoclastogenesis the bones were decalcified in 10% EDTA in PBS pH 7.4.The decalcified femurs were mounted in the cryo-sectioning mounting media (Electron Microscopy Sciences, Hatfield, PA) and stored at −80°C before sectioning. The 5–6 µm sections were taken and fixed in 10% formalin with PBS for 30 minutes, washed three times with PBS, blocked with 2% serum in PBS for 15 minutes. The bone sections were immunostained with Tartrate Resistant Acid Phosphatase (TRAP) antibody (Santa Cruz Biotechnology, Inc., Santa Cruz, CA) overnight at 4°C. The sections were washed three times with PBS and incubated with Fluorescein conjugated secondary antibody. The sections were washed three times and mounted in DAPI containing media. The fluorescence images were taken with Carl Zeiss inverted fluorescence microscope.

Bone marrow derived macrophages (BMDMs) were prepared from whole bone marrow of mice and cultured as follows. Bone marrow (BM) cells from tibia and femur of 6- to 10-week-old mice were flushed out with ice-cold α-MEM containing 10% heat-inactivated FBS and penicillin/streptomycin. The cell suspension was layered onto a Ficoll-Paque layer and centrifuged to buoyancy separate the monocytes (GE Healthcare Bio-Sciences Corp., NJ). This monocyte layer was washed and cultured with α-MEM and 10% FBS for 2–12 hr. The non-adherent cells were maintained with RANKL (100 ng/mL) and M-CSF (50 ng/mL) for 3 days, when new media was added to induce cells to undergo OC differentiation.

### Osteoclastogenesis cytokine sensitivity

The concentration of RANKL was progressively increased, (12.5, 25, 50 and 100 ng/ml), but not M-CSF (2.5 ng/ml). The level of the invariant cytokine is that used in our standard osteoclastogenic assay is stated below. After 9 days, TRAP-expressing multi-nucleated OCs was scored.
